# The roles of teacher and parental support on adolescent cyber-bystander behaviors: a path analysis

**DOI:** 10.3389/fpsyg.2024.1374071

**Published:** 2024-05-22

**Authors:** Qiqi Chen, Qianru Wu

**Affiliations:** Department of Applied Social Sciences, The Hong Kong Polytechnic University, Kowloon, Hong Kong SAR, China

**Keywords:** cyberbullying, bystander, empathy, internet self-efficacy, teacher support

## Abstract

**Introduction:**

The role of bystanders in cyberbullying situations is critical, with the potential to significantly influence outcomes. Bystanders who demonstrate positive behaviors—such as reporting incidents or supporting victims—can help to mitigate the damaging effects of cyberbullying. Based on the Social Cognitive Theory, this study seeks to address the psychosocial mechanisms that underlie positive cyber-bystander behaviors.

**Methods:**

A total of 1,716 students in Grades 8–12 from three secondary schools in China participated in this study. Path analysis was utilized to delineate the relationships between internet self-efficacy, empathy, teacher and parental support, and cyber-bystander behaviors.

**Results:**

Adolescents who received greater support from teachers were more likely to demonstrate increased internet self-efficacy and empathy. In contrast, higher levels of parental warmth were associated with lower levels of internet self-efficacy. Teacher support and parental warmth exerted an indirect effect on positive bystander behaviors through empathy.

**Discussion:**

The importance of parental warmth paired with Internet self-efficacy in preventing online interpersonal violence and motivate active bystander behaviors is considerable. We recommend adopting a nuanced approach that differentiates between empathy and internet self-efficacy in cyber-bystander research.

## 1 Introduction

### 1.1 Cyberbullying and cyber-bystander behaviors

With the rapid rise of the Information Communication Technologies (ICTs), adolescents nowadays are able to access information and form virtual relationships online. However, frequent online communication may also considered as an exposure factor that increases the risk of being victimized in cyberspace, such as leakage of personal information, online harassment and cyberbullying (Hong et al., 2016; Ngo et al., 2020). Cyberbullying refers to as “the behavior performed through electronic or digital media by individuals or groups that repeatedly communicates hostile or aggressive messages intended to inflict harm or discomfort on others” (Tokunaga, 2010). Similarly to the negative consequences of offline bullying, victims of cyberbullying are at great risk of experiencing a myriad of mental health problems, including depression, phobic anxiety, suicidal ideation, substance use and low self-esteem (Kowalski et al., 2014; Wang, 2021). In fact, previous studies have argued that the negative outcome of cyberbullying may be more detrimental than traditional bullying due to the nature of the Internet, which seemingly leaves the victim no escape and the harmful materials was permanently preserved and easily spread (Shultz et al., 2014).

Whereas cyber-bystanders, representing the potentially unlimited third-party participants are considered crucial for combatting cyberbullying by asserting their influence over the course of cyberbullying (Wang, 2021). The publicity of cyberspace make adolescents more susceptible to exposure to cyberbullying as bystanders, but it also constrains their ability to react when witnessing cyberbullying, even if it sometimes only requires clicking a button (Bastiaensens et al., 2014). In fact, bystander reactions can either enhance or mitigate the harmful consequences of cyberbullying depending on whether they proactively take actions to intervene in such incidents (DeSmet et al., 2016). For instance, with the desire to affiliate with the “stronger” party, cyber-bystanders might choose to support the perpetrator by forwarding hurtful posts or even just by clicking the LIKE button (Olenik-Shemesh et al., 2017). Therefore, understanding bystanders’ behavior patterns in cyberbullying is crucial for designing targeted interventions that encourages positive responses.

### 1.2 The roles of empathy and self-efficacy

Social Cognitive Theory provides a theoretical framework for understanding, predicting, and changing human behavior, which highlights the need to consider the interaction of personal, behavioral, and environmental factors together influencing one’s behavior (Bandura, 1986). This theory has been widely adopted in previous research to explain and reduce the episodes of traditional bullying and cyberbullying (Wachs et al., 2020). For example, it was found that bystanders who often defend others typically display strong moral compasses, high senses of responsibility, social self-efficacy, and empathy, and also tend to have close relationships with victims and enjoy high status among peers (Patterson et al., 2017). Based on the Social Cognitive theory and prior research on factors affecting cyberbullying bystander behaviors, the present study focus on empathy and Internet self-efficacy as the personal factors, and teacher support and parental warmth as the environmental factor influencing positive adolescent cyber-bystander behaviors.

Empathy is generally described as consisting of two major constructs, affective empathy and cognitive empathy (Graf et al., 2019). The former describes the ability to experience and share the emotion of others, while the latter refers to the ability to understand the emotion (including motivational, intentional and self-regulative experience) of others (Graf et al., 2019). Empathic experience, therefore, is considered a result of both affective and cognitive empathy. It is also indicated as one of the mechanisms of prosocial behavior or altruism, which was typically found to be positively associated with reduced bullying and cyberbullying behaviors (Schultze-Krumbholz et al., 2016). Researchers (Barlińska et al., 2018) also investigated the differential effect of affective empathy and cognitive empathy with experiments in stimulate adolescent cyber-bystander behaviors and revealed that cognitive empathy activation significantly increases the likelihood of positive bystander behaviors.

Empathetic individuals who has better perspective-taking skills also better at understanding the distress of the victim, thus are more inclined to intervene and provide emotional support to the victim (DeSmet et al., 2018). In terms of bystander reactions, successful empathy activation could diminish the likelihood of negative cyber-bystander behavior, by not supporting the perpetrators. Thus, given that empathy is a potential inhibitor against passive bystander behavior, determining whether education or resources can effectively enhance the empathetic capacity of targeted groups is crucial to success. We hypothesize that (H1) higher level of empathy is significantly related to positive cyber-bystander behaviors.

Personal agency is another key factor of Social Cognitive Theory when it relates to defending behavior, which refers to an individual’s ability to develop and direct their actions toward a task (Clark and Bussey, 2020). Self-efficacy plays an critical role in personal agency, which originally refers to the belief in one’s ability to perform an activity in a specific context (Bussey et al., 2020). The notion of Internet self-efficacy describes an individual’s belief in their ability to accomplish tasks across the online realm and effectively use digital technologies to protect their privacy and avoid online risks (Torgal et al., 2023). Prior research on cyberbullying has revealed that individuals who perceive themselves as lacking the ability to control their Internet-related behaviors found cyberbullying incidents anxiety-provoking and were less willing to take action when they witnessed one online (Domínguez-Hernández et al., 2018). In contrast, those with high Internet self-efficacy are more likely to take proactive actions such as reporting bullying incidents or offering help to the victims (Hinduja and Patchin, 2010). Therefore, when witnessing others facing difficult situations online, they are often more skillful and confident in resolving conflicts, providing assistance to the victim, and rallying support against the perpetrators (Pellas, 2014). Thus, we hypothesize that (H2) higher level of Internet self-efficacy is significantly related to positive cyber-bystander behaviors.

### 1.3 The roles of parental and teacher support

Social support as a key environmental factor may act as a buffer against cyberbullying and associated negative experiences (Olenik-Shemesh et al., 2017). Specifically, family and teacher support play an important role in helping adolescents coping with cyberbullying and also provided a channel for them to seek help (Elsaesser et al., 2017; Grunin et al., 2021). The differentiated impacts of bystander reactions was also found to be mediated by the social support and emotional comfort they receive from others (Gradinger et al., 2009). Although most cyberbullying incidents happen outside of school, the harmful impacts that students are undergoing can largely affect their behaviors and experiences at school (Eden et al., 2013). Teacher support, which encompasses both emotional and instructional assistance, is significant in shaping students’ reactions to cyberbullying (Sulkowski and Simmons, 2018). Teachers can provide assistance through immediate care and consistent responses. In terms of early intervention, teachers can also foster a supportive environment that encourages positive bystander behaviors by promoting empathy and teaching effective internet use (Tokunaga, 2010). Additionally, they can provide guidance on how to respond to cyberbullying incidents and encourage students to report such incidents (Longobardi et al., 2018). Therefore, teacher support can contribute to a harmonious school culture where cyberbullying is not tolerated and positive bystander behaviors are encouraged. Therefore, we hypothesize that (H3) teacher support is significantly related to positive cyber-bystander behaviors.

Previous studies on parenting style also found that an indulgent parenting style characterized by the use of reasoning and warmth can serve as a protective factor against both bullying and cyberbullying victimization (Martínez et al., 2019). Emotional warmth provided by parents and harmonious family relationships can set an example of interpersonal interaction for adolescents to help them to build and maintain positive peer relationships (Chen et al., 2020). Children raised in supportive environments may replicate their experiences at home and actively defend the victims in bullying situations (Evans and Smokowski, 2016). These warm and supportive households often foster empathy, altruism and instill moral values, therefore encourage adolescents to stand up against cyberbullying and support their peers. Researchers (Olenik-Shemesh et al., 2017) explored personal-socio-emotional differences between active and passive cyber-bystanders and they found that active bystanders reported having significantly more social support from significant others than their counterparts in the passive group. Review studies have further revealed that anti-cyberbullying programs may be benefit from integrating parent trainings on positive parenting skills and promoting open parent–child communications (Nocentini et al., 2015; Chen et al., 2021). Thus, we hypothesize that (H4) parental warmth is significantly related to positive cyber-bystander behaviors.

When discussing the effects of teacher support and parental warmth on positive cyber-bystander behavior, it is essential to consider the role of personal factors in influencing their actions. Specifically, not all individuals with high teacher support or parental warmth necessarily engage in helping behaviors online. Their levels of empathy or their perceived Internet-self-efficacy may also influence how they would intervene when witnessing cyberbullying. Before helping the victim, bystanders would assess their ability to intervene and determine an appropriate way of helping. Self-efficacy plays a significant role in this decision-making process (Huang et al., 2019). When bystanders perceive they have affirmative judgments of their Internet skills and heightened sense of control over the virtual environment, they are more likely to offer help to the victim since they are confident in achieving desired outcome through online actions (Hu et al., 2023).

### 1.4 Current study

Parents as the central and closest figure in a child’s life, has a significant influence on the development of empathy in children through socialization and caring behavior (Abdullah and Salim, 2020). When individuals have dysfunctional relationships with their parents, their development of empathy may reduce, which in turn resulting in a potential decline in helping behaviors (Zhao et al., 2023). On the other hand, teacher’s awareness and commitment to support can reduce bullying and appeal to education systems to consider cyberbullying when developing educational programs. By promoting empathy and cultivating Internet self-efficacy at school, adolescents may become more skillful and willing to offer help as cyber-bystanders. Together, we propose that the interplay of parental and teacher support significantly influences positive cyberbullying bystander behaviors by promoting positive psychosocial characteristics. By understanding these dynamics, we can develop effective strategies to combat cyberbullying and promote positive online interactions. Therefore, drawing on the Social Cognitive Theory and relevant prior research, we hypothesize that (H5) Internet self-efficacy mediates the relationship between parental warm and positive cyber-bystander behaviors; (H6) Internet self-efficacy plays a mediating role between teacher support and positive bystander behaviors; (H7) empathy plays a mediating role between parental warmth and positive bystander behaviors; and (H8) empathy plays a mediating role between teacher support and positive bystander behaviors.

## 2 Methods

### 2.1 Study design and procedure

This study involved students aged 13–18 years from grades 8–12, drawn from three middle schools in Qingdao and Wuhan, China. The cities were chosen for their status as large, economically and culturally influential urban centers in China, which play a significant role in shaping regional trends in adolescent behavior. Data collection occurred in the fall semester of 2022, using a convenience sampling method. Initially, approximately 2,400 students from the three schools were invited to participate in the study. Ultimately, the study included 1,716 adolescents, achieving a response rate of 71.5%. Of these, 1,132 (66.0%) were from public schools and 584 (34.0%) from vocational schools, 764 participants (44.52%) were boys. The average age of the participants was 14.60 years (SD = 1.35). All students and their parents provided informed consent prior to participating in the survey. A trained research assistant asked the students to fill out a web-based self-report questionnaire during school hours, ensuring sufficient separation in the classroom to maintain privacy. The survey took about 30 min to complete. The study received ethics approval from the Institutional Review Board of the researchers’ affiliated University.

### 2.2 Measures

#### 2.2.1 Positive bystander behaviors

We constructed nine possible cyberbullying bystander behaviors with reference to the categories of coping in previous literature (Raskauskas and Huynh, 2015), including “wait and see,” “seek the truth,” “retaliate online,” “forward and spread,” “tip off,” “engage in bullying,” “ask for help,” “call the police,” and “pay no attention.” Participants were asked to select all the responses that applied to them in witnessing cyberbullying scenarios. Specifically, we assessed participants’ reactions as bystanders to various cyberbullying victims based on their roles and subjective attitudes toward these victims. The victims could include family members, schoolmates, teachers, and public figures. In this study, we included four of the behaviors as positive behaviors, including “seek the truth,” “tip off,” “ask for help,” and “call the police.” The survey items were multiple-choice, allowing participants to select all responses that applied to them. For each scenario, participants could potentially score 1 for each positive behavior they chose to engage in. The results were recoded as 1 (yes) if participants selected such a case, and as 0 (no) if they did not, with higher scores indicating a greater likelihood of choosing these bystander behaviors. The total score for each behavior across all scenarios was then calculated for each participant. The Cronbach’s alpha for the positive bystander behaviors in this study was 0.93.

#### 2.2.2 Teacher support

Participants’ perceived teacher support was examined with the 7-item teacher-to-student subscale from the School Climate Measure (Jia et al., 2009). Example items in this subscale was “The teachers believe I can do well.” All the seven items were rated on a 4-point Likert scale (1 = never, 4 = always), with higher scores indicating higher levels of perceived support from teachers at school. The subscale demonstrated good reliability with a Cronbach’s α 0.95 in current study.

#### 2.2.3 Parental warmth

Parental warmth was measured using the 7-item Emotional Warmth subscale from the short-form Egna Minnen and Barndoms Uppfostran for the Chinese scale (s-EMBU-C; Jiang et al., 2010), which was designed to measure perceived parental rearing behaviors. Example items in this subscale was “My parents try to encourage me to become the best.” Each item was rated on a 4-point Likert scale (1 = “never,” 4 = “always”). The subscale showed good reliability with a Cronbach’s α 0.92.

#### 2.2.4 Empathy

The Basic Empathy Scale (BES; Sánchez-Pérez et al., 2014) was used to assess the participants’ empathy levels. This scale consists of 20 items that gauge cognitive and affective empathy, asking children to express their level of agreement with each statement. Experienced bilingual social workers translated the scale from English to Chinese and then back to English using the forward-backward method (Brislin, 1970). This process ensured the accuracy of the translation by comparing it with the original version. Each item on the scale was scored using a five-point Likert scale, ranging from 1 (“Strongly disagree”) to 5 (“Strongly agree”). The overall empathy score was computed by summing up the responses to all items. The BES scale demonstrated good reliability, with an overall Cronbach’s α of 0.84, 0.82 for affective empathy, and 0.80 for cognitive empathy.

#### 2.2.5 Internet self-efficacy

The participants’ perceived confidence in performing internet activities was gauged using the 17-item Internet Self-Efficacy Scale (ISS; Kim and Glassman, 2013). The ISS captures a variety of internet self-efficacy related activities: Reactive (6 items), Differentiation (4 items), Organization (3 items), Communication (2 items), and Search Self-Efficacy (2 items). Example items are “I can effectively use social networking sites to connect with others,” and “I can find valuable information about important topics on the Internet.” Participants were asked to rate on a 7-point Likert scale, where 1 represented “not at all confident” and 7 denoted “very confident.” In this study, the ISS demonstrated excellent reliability, with an overall Cronbach’s α of 0.97 and high Cronbach’s α for each subscale: Reactive (0.93), Differentiation (0.92), Organization (0.93), Communication (0.86), and Search Self-Efficacy (0.91).

### 2.3 Statistical analysis

This study aimed to compare the effects of teacher support at school and parental warmth at home on adolescents’ positive cyberbullying bystander behaviors, specifically focusing on the roles of empathy and internet self-efficacy. To achieve these objectives, we calculated the means and standard deviations (SD) of the total scores for the outcome variables and stratified them by gender. Gender differences were examined using t-tests. A correlation matrix was constructed to illustrate the relationships among all variables. Path analyses were conducted to assess the influence of teacher support, parental warmth, empathy, and internet self-efficacy on the prediction of positive bystander behaviors, with adjustments for the covariates of adolescent age and gender. All statistical significance was two-tailed, and significance was set at the *p* < 0.05 level. All the above analyses were performed using Stata version 18.0. Model estimation was checked using Mplus 8.0, employing the maximum likelihood robust (MLR) estimator to address potential non-normality in the data, and the MODEL CONSTRAINT syntax to check the indirect effects (Muthén and Muthén, 2015).

## 3 Results

### 3.1 Descriptive statistics of the outcome variables

Table 1 presents the scores of the variables, compared by gender. Boys reported significantly higher levels of perceived teacher support (*t* = 2.82, *p* < 0.01) than girls did. Conversely, girls demonstrated higher levels of empathy (*t* = 9.09, *p* < 0.001) and the overall positive bystander behaviors (*t* = 3.17, *p* < 0.01) than boys. No significant difference was found between boys and girls in terms of internet self-efficacy, parental warmth, and some positive bystander behaviors, such as “ask for help” and “seeking the truth.”

**Table 1:** Outcome variables of participants by gender (*N* = 1,716).

M (SD)	W^a^	Total (*N* = 1,716)	Boys (*n* = 764)	Girls (*n* = 952)	t	Cohen’s d
Internet self-efficacy	0.97***	43.71 (18.18)	44.15 (18.39)	43.36 (18.02)	0.89	0.04
Empathy	0.98***	54.78 (5.87)	53.38 (5.64)	55.91 (5.80)	9.09***	0.44
Teacher support	0.93***	20.93 (4.61)	21.28 (4.84)	20.65 (4.41)	2.82**	0.14
Parental warmth	0.97***	19.78 (5.21)	19.98 (5.37)	19.63 (5.08)	1.38	0.07
Positive bystander	0.97***	12.27 (8.00)	11.59 (8.12)	12.82 (7.87)	3.17**	0.15
Seek the truth	0.96***	2.51 (2.87)	2.43 (2.58)	2.58 (2.83)	1.13	0.06
Tip off	0.97***	3.14 (3.01)	2.92 (3.00)	3.31 (3.01)	2.67**	0.13
Ask for help	0.97***	2.91 (2.80)	2.81 (2.87)	2.98 (2.74)	1.25	0.06
Call the police	0.98***	3.72 (2.97)	3.43 (3.03)	3.95 (2.89)	3.63***	0.18

*p < 0.05, **p < 0.01, ***p < 0.001.^a^W by Shapiro–Wilk test.

Table 2 displays the correlations between all outcome variables. All variables were positively related to each other (rs range from 0.11 to 0.48, ps < 0.001), with the exception of internet self-efficacy, which did not have a significant relationship with either parental warmth or positive bystander behavior.

**Table 2:** Correlations among variables.

	Variables	1	2	3	4	5
1	Internet self-efficacy	1.00				
2	Empathy	0.15***	1.00			
3	Teacher support	0.11***	0.20***	1.00		
4	Parental warmth	0.01	0.21***	0.48***	1.00	
5	Positive bystander	0.04	0.24***	0.21***	0.23***	1.00

***p < 0.001.

### 3.2 Path analysis

**Figure 1:**
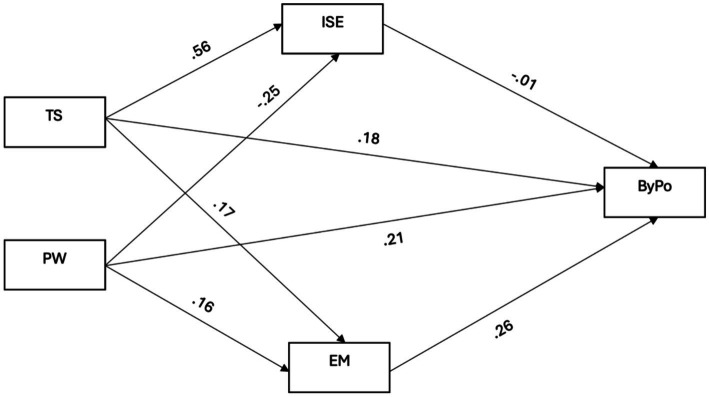
Effects of teacher support and parental warmth on positive bystander behaviors through internet self-efficacy and empathy. Adolescent’s age and gender are used as covariates. TS, Teacher support; PW, Parental warmth; ISE, Internet self-efficacy; EM, Empathy; ByPo, Positive bystander behaviors.

As depicted in Figure 1 and Table 3, the path analysis results indicate that teacher support (*B* = 0.18, *p* < 0.001), parental warmth (*B* = 0.21, *p* < 0.001), and empathy (*B* = 0.26, *p* < 0.001) were significantly related to positive bystander behaviors. Adolescents with higher levels of teacher support exhibited higher odds of internet-self-efficacy (*B* = 0.56, *p* < 0.001) and empathy (*B* = 0.17, *p* < 0.001). Conversely, higher levels of parental warmth were found to be negatively related to internet self-efficacy (*B* = −0.25, *p* < 0.05).

**Table 3:** Direct and indirect effects of teacher support and parental warmth on positive bystander behaviors through internet self-efficacy and empathy.

B (95% CI)		
*Direct paths*	Positive bystander behaviors	
Teacher support	0.18*** (0.09, 0.27)	
Parental warmth	0.21*** (0.13, 0.29)	
Internet self-efficacy	−0.01 (−0.02, 0.01)	
Empathy	0.26*** (0.19, 0.32)	
	Internet self-efficacy	Empathy
Teacher support	0.56*** (0.35, 0.77)	0.17*** (0.10, 0.24)
Parental warmth	−0.25* (−0.43, −0.06)	0.16*** (0.10, 0.22)
Indirect paths		
Teacher support → ISE → ByPo	−0.01 (−0.01, 0.01)	
Teacher support → EM → ByPo	0.05*** (0.02, 007)	
Parental warmth → ISE → ByPo	0.01 (−0.01, 0.01)	
Parental warmth → EM → ByPo	0.04*** (0.02, 006)	

*p < 0.05, ***p < 0.001.ISE, Internet self-efficacy; EM, Empathy; ByPo, Positive bystander behaviors.

The pathway model results also showed small but significant indirect effects on positive bystander behaviors (ps < 0.001). Specifically, teacher support had an indirect effect on positive bystander behaviors through empathy (*B* = 0.05, *p* < 0.001), and parental warmth had an indirect effect on positive bystander behaviors through empathy (*B* = 0.04, *p* < 0.001).

## 4 Discussion

### 4.1 Descriptive results and gender differences in outcome variables

The current study examined the effects of teacher support and parental warmth on adolescents’ positive cyber-bystander behaviors, taking the roles of empathy and Internet self-efficacy into consideration. When analyzing the results by gender, it was found that boys reported significantly higher levels of perceived teacher support than girls, whereas girls reported significantly higher levels of empathy and positive cyber-bystander behaviors than boys. These results were in accordance with previous gender differences studies, which revealed that girls were more willing to intervene while boys tended to remain passive when witnessing cyberbullying (Wang and Kim, 2021). Moreover, this results may be attributed to higher empathy reported by female students, which in turn lead to a greater inclination to help the victim (Allison and Bussey, 2017; Hayashi et al., 2023). In terms of Internet self-efficacy, our results diverge from previous research on other Internet-related risky behaviors. Contrary to studies where male students reported better Internet self-efficacy than female students—often attributed to males being more experienced with Internet use and females demonstrating a less positive attitude and greater anxiety toward the Internet (Hu et al., 2012)—we found no significant difference between boys and girls. This could be attributed to changes in societal norms and educational practices that promote equal access to technology for both genders in this digital age. Additionally, increased awareness and efforts to bridge the digital divide may have contributed to leveling the playing field. Future studies might explore these generational variations and individual Internet experiences influenced by factors such as socioeconomic status, parental involvement, or personal interests.

The results of the correlation analysis demonstrated that empathy, teacher support, and parental warmth were positively related with positive cyber-bystander behaviors, which supported our hypothesis 1, 3, and 4. These results were coincide with previous studies, highlighting the role of supportive relationships and empathy in promoting prosocial behaviors (DeSmet et al., 2014). Teacher support was related to the overall positive climate at school, and higher perceived school and teacher support can increase students’ willingness to report and seek help in cyberbullying cases (Schultze-Krumbholz et al., 2020). With regards to parental warmth, when parents react supportively to adolescents’ self-disclosure of cyber-bystander experience, they are more confident in standing up on behalf of the victim (Levy and Sela-Shayovitz, 2020). Lastly, empathy as a potential important inhibitor of passive bystander behaviors which requires high levels of emotional-experiencing and perspective-taking, can limit the support for cyberbullies (Panumaporn et al., 2020).

### 4.2 Relationship between internet self-efficacy and cyber-bystander behaviors

However, we did not yield significant results to support our hypothesis 2, since we did not observe significant correlations between Internet self-efficacy and positive cyber-bystander behaviors. One possible explanation for this is that Internet self-efficacy may not directly translate into helping behaviors itself, but it may assert its influence through other indirect pathways, such as moral judgments or empathy. Researchers (Hu et al., 2023) investigated the association of empathy, Internet moral judgment, and Internet self-efficacy with bystander helping behavior among adolescents. The authors found that empathy was positively related to positive bystander behavior, and Internet moral judgments mediated this relationship. Moreover, Internet self-efficacy moderated the latter half of the mediation pathway, which means that Internet self-efficacy may only enhance the effect of moral judgment and active bystander behavior in cyberbullying, but not necessarily directly increase helping behavior *per se*. Another plausible reason for this might be that Internet self-efficacy may have different effects on different types of bystander behaviors in cyberbullying. Researchers found that participants who had cyberbullying experience or knew of cyberbullying happening to friends would intervene more than those who did not had such experiences, while when cyberbullying was happening to the general public or strangers, they tended to ignore the incidents (Panumaporn et al., 2020). This may suggest that Internet self-efficacy may be associated with active cyber-bystander behaviors only under certain circumstances, such as when the bystanders had been cyberbullied before or they have close relationship with the victim. Thus, future studies examining the mediating role of other related factors (e.g., previous cyberbullying experience and relationship with the victim) on the relationship between Internet-self efficacy and active bystander behavior is needed.

### 4.3 Relationships between parent and teacher support and cyber-bystander behaviors

Notably, we found that higher levels of parental warmth were negatively related to Internet self-efficacy. This result was inconsistent with previous findings suggested that parental warmth and support enhances children’s self-efficacy (Aumeboonsuke, 2017). Although this appears counter-intuitive, this finding may be due to the unique influence of parental warmth on domain specific self-efficacy. In this case, we are interested in how parental warmth may affect adolescents’ Internet-related self-efficacy. Parental warmth may be accompanied by enhanced parental monitoring in terms of Internet use, which potentially limit adolescents’ exposure and access to the Internet, especially those that are considered by parents as inappropriate for children (Chou and Lee, 2017). More specifically, authoritative parents that characterized by high level of warmth and control, not only participate in co-viewing with their children more often, but also tended to restrict their children’s time spent online and use technologies to block undesired contents than neglectful parents who exhibit both low warmth and control. Therefore, this “over-protection” comes with parental warmth may limits adolescents’ ability of acquiring Internet-related knowledge’s or skills, resulting in lowered Internet self-efficacy in children. Thus, future studies may delve into more depth in terms of different Internet parenting styles that captures more aspects of parenting strategies and examining its impact on children’s Internet self-efficacy.

### 4.4 The mediating role of empathy and internet self-efficacy

With regards to the results of path analysis, we examined the direct and indirect effects of teacher support and parental warmth on positive bystander behaviors through Internet self-efficacy and empathy. The model demonstrated several significant direct effects. Specifically, teacher support had direct, positive effects on Internet self-efficacy, empathy and positive cyber-bystander behaviors. Empathy had direct and positive effect on positive bystander behaviors. Furthermore, parental warmth had direct, positive effects on positive cyber-bystander behaviors and empathy, but manifested negative effects on Internet self-efficacy. As we discussed before, the negative relationship between parental warmth and Internet self-efficacy might simply implies the aspects of heightened parental control and monitoring, which may limit adolescents’ autonomy and exploration on the Internet. But if we explore the influence on positive bystander behaviors, a warm parent–child relationship can also provide guidance and support to the children on healthy Internet use. In addition, warm and responsive family climate provide adolescents with a context to feel safe and to process difficult emotions, reducing involvement in risky behaviors (Elsaesser et al., 2017). Furthermore, knowing effective and assertive strategies as well as support resources are crucial in motivating bystanders to proactively intervene in cyberbullying cases, especially asking adults for help that resolves the problem (Domínguez-Hernández et al., 2018). Researchers stated that these essential skill sets and emotional resources are considered essential for bystanders to take positive actions (DeSmet et al., 2016). Therefore, the importance of parental warmth paired with Internet self-efficacy in preventing online interpersonal violence and motivate active bystander behaviors is considerable.

The proposed model also reflected significant indirect effects, where both teacher support and parental warmth showed small but significant indirect effects on positive bystander behaviors through empathy. Our results of the indirect effects generated insights that underscore the complex interplay of these factors. We found a significant mediating role of empathy affecting the relationship between teacher support on positive bystander behaviors. It means that teacher support not only directly associated with active bystander actions in cyberbullying, but also indirectly associated with helping behaviors of adolescents through empathy. To help students to cultivate a greater empathy, stronger relationships and effective communication at school is crucial to induce them to take on another person’s perspective (Altavilla et al., 2021). In terms of teacher-student relationships, emotional experiences and the acquisition of knowledge in daily interactions plays a fundamental role in the construction of socio-emotional competence and in the development of empathy (Altavilla et al., 2021). It is therefore required for teachers to have a strong educational sensitivity to recognize an effective empathic educational approach. Researchers (Waters et al., 2020) proposed that by instilling moral attitude and civic virtues, such as responsibility, respect, caring, fairness, empathy and trustworthiness, in hopes that students will understand and intrinsically make good decisions when encountering or witnessing cyberbullying online. Future research may keep on examining whether various types of teacher support (e.g., emotional, informational or instrumental) have differentiated impact on the relationship between empathy and positive cyber-bystander behavior. By doing so, we are hoping to design more targeted intervention or education programs that encourages adolescents exhibiting more active bystander behaviors in the face of cyberbullying.

### 4.5 Limitations

Finding of the current study highlights the multifaceted nature of these behaviors and the importance of holistic interventions. While this study uncovers important findings, it also presents several limitations. Firstly, the responses were collected using self-report measures that rely on participants’ perceptions of their empathy, internet self-efficacy, and cyberbullying experiences. Social desirability bias may have led participants to provide overly positive responses. The reliance on self-reported data introduces potential inaccuracies due to subjective perceptions and memory biases. There could also be a discrepancy between reported supportive attitudes and the lower frequency of actual positive bystander behaviors, indicating a gap between belief and action. Additionally, while our large sample size lends robustness, the generalization to other demographics may be constrained, and non-normal data distribution could impact the analysis outcomes despite statistical justifications. Future studies could include objective assessments such as behavioral observations or third-party evaluations to provide a more comprehensive understanding of these traits. Secondly, the evidence from path analysis in a cross-sectional study may prevent the drawing of causal inferences from these findings. Therefore, future studies using experimental or longitudinal designs are encouraged to examine the long-term influences or causal effects of the variables of interest and their intermediary effects. Thirdly, there could be other unmeasured variables that influence the relationships, such as Internet moral judgment, personal resilience and previous victimization experiences. These factors could potentially play a more significant role in changing the dynamics of the associations than the tested parental and teacher support in cyberbullying experiences.

### 4.6 Implications

This study underscores the intricate network of factors that influence adolescent cyber bystander behaviors. Our results may provide implications for both theoretical and practical advancement by demonstrating the complex roles that empathy and internet self-efficacy play in facilitating positive bystander behaviors. While social capital and self-efficacy are important individual factors for child development in the digital era, studies have indicated that excessive unguided internet use can lead to negative consequences, such as internet addiction and social alienation (Sigerson et al., 2017; Tzavela et al., 2017). Interestingly, our study also highlights that internet self-efficacy alone does not directly correlate with positive bystander behaviors, indicating that other factors might influence this behavior. This observation opens up new avenues for research to explore additional variables that could mediate or moderate the relationship between internet self-efficacy and bystander actions. Identifying these factors could lead to more targeted cyberbullying prevention strategies that address these nuanced elements of bystander behavior. Future research should delve deeper into these relationships, considering other potential moderating and mediating factors in family and school environments, and cultural contexts. Moreover, these findings have significant implications for collaborations between educators and parents, highlighting the importance of fostering supportive environments to promote positive bystander behaviors among adolescents. Specifically, the findings from our study have significant implications for emphasizing the crucial role of supportive environments in both school and home settings for addressing cyberbullying. The positive impact of teacher support and parental warmth on bystander behaviors suggests that interventions aimed at enhancing these relationships can be effective in promoting active intervention among bystanders in cyberbullying situations. Schools and families should consider implementing programs that foster stronger relationships and communication skills, which could empower students to take action when witnessing cyberbullying. Programs in schools may consider developing platforms that make tasks more engaging and intuitive, or creating educational and training strategies that increase parental involvement in these tasks (Kim and Glassman, 2013). This could also involve providing counseling or therapy to address the emotional impact of cyberbullying, as well as education and training on responsible online behaviors. Overall, these intricate findings emphasize the need for a more nuanced approach to provide comprehensive, individualized services in cyberbullying prevention and adolescent mental health.

## Data availability statement

The raw data supporting the conclusions of this article will be made available by the authors, without undue reservation.

## Ethics statement

The studies involving humans were approved by the Institutional Review Board of the Hong Kong Polytechnic University. The studies were conducted in accordance with the local legislation and institutional requirements. Written informed consent for participation in this study was provided by the participants' legal guardians/next of kin.

## Author contributions

QC: Conceptualization, Formal analysis, Funding acquisition, Methodology, Project administration, Writing – original draft, Writing – review & editing. QW: Writing – original draft, Writing – review & editing.
